# O1.3. A COMPUTATIONAL TRIAL-BY-TRIAL EEG ANALYSIS OF HIERARCHICAL PRECISION-WEIGHTED PREDICTION ERRORS

**DOI:** 10.1093/schbul/sby015.185

**Published:** 2018-04-01

**Authors:** Sara Tomiello, Dario Schöbi, Lilian Weber, Helene Haker, Iglesias Sandra, Klaas Enno Stephan

**Affiliations:** 1 ETH Zurich; 2 University of Zurich & ETH Zurich

## Abstract

**Background:**

Action optimisation relies on learning about past decisions and on accumulated knowledge about the stability of the environment. In Bayesian models of learning, belief updating is informed by multiple, hierarchically related, precision-weighted prediction errors (pwPEs). Recent work suggests that hierarchically different pwPEs may be encoded by specific neurotransmitters such as dopamine (DA) and acetylcholine (ACh). Abnormal dopaminergic and cholinergic modulation of N-methyl-D-aspartate (NMDA) receptors plays a central role in the dysconnection hypothesis, which considers impaired synaptic plasticity a central mechanisms in the pathophysiology of schizophrenia.

**Methods:**

To probe the dichotomy between DA and ACh and to investigate timing parameters of pwPEs, we tested 74 healthy male volunteers performing a probabilistic reward associative learning task in which the contingency between cues and rewards changed over 160 trials between 0.8 and 0.2.

Furthermore, the current study employed pharmacological interventions (amisulpride / biperiden / placebo) and genetic analyses (COMT and ChAT) to probe DA and ACh modulation of these computational quantities. The study was double-blind and between-subject.

We inferred, from subject-specific behavioural data, a low-level choice PE about the reward outcome, a high-level PE about the probability of the outcome as well as the respective precision-weights (uncertainties) and used them, in a trial-by-trial analysis, to explain electroencephalogram (EEG) signals (64 channels). Behavioural data was modelled implementing three versions of the Hierarchical Gaussian Filter (HGF), a Rescorla-Wagner model, and a Sutton model with a dynamic learning rate. The computational trajectories of the winning model were used as regressors in single-subject trial-by-trial GLM analyses at the sensor level. The resulting parameter estimates were entered into 2nd-level ANOVAs. The reported results were family-wise error corrected at the peak-level (p<0.05) across the whole brain and time window (outcome phase: 0 - 500ms).

**Results:**

A three-level HGF best explained the data and was used to compute the computational regressors for EEG analyses. We found a significant interaction between pharmacology and COMT for the high-level precision-weight (uncertainty). Specifically:

- At 276 ms after outcome presentation the difference between Met/Met and Val/Met was more positive for amisulpride than for biperiden over occipital electrodes.

- At 274ms and 278 ms after outcome presentation the difference between Met/Met and Val/Met was more negative over fronto-temporal electrodes for amisulpride than for placebo, and for amisulpride than for biperiden, respectively.

No significant results were detected for the other computational quantities or for the ChAT gene.

**Discussion:**

The differential effects of pharmacology on the processing of high-level precision-weight (uncertainty) were modulated by the DA-related gene COMT.

Previous results linked high-level PEs to the cholinergic basal forebrain. One possible explanation for the current results is that high-level computational quantities are represented in cholinergic regions, which in turn are influenced by dopaminergic projections. In order to disentangle dopaminergic and cholinergic effects on synaptic plasticity further analyses will concentrate on biophysical models (e.g. DCM). This may prove useful in detecting pathophysiological subgroups and might therefore be of high relevance in a clinical setting.

